# Incidence of thromboembolism in patients with COVID-19: a systematic review and meta-analysis

**DOI:** 10.1186/s12959-020-00248-5

**Published:** 2020-11-23

**Authors:** Kochawan Boonyawat, Pichika Chantrathammachart, Pawin Numthavej, Nithita Nanthatanti, Sithakom Phusanti, Angsana Phuphuakrat, Pimjai Niparuck, Pantep Angchaisuksiri

**Affiliations:** 1 Department of Medicine, Faculty of Medicine Ramathibodi Hospital, Mahidol University, Bangkok, Thailand; 2 Department of Clinical Epidemiology and Biostatistics, Faculty of Medicine Ramathibodi Hospital, Mahidol University, Bangkok, Thailand; 3 Chakri Naruebodindra Medical Institute, Faculty of Medicine Ramathibodi Hospital, Mahidol University, Samut Prakan, Thailand

**Keywords:** COVID-19, Venous thromboembolism, Arterial thromboembolism, Meta-analysis

## Abstract

**Background:**

Since the beginning of the coronavirus disease 2019 (COVID-19) pandemic, the incidence of thromboembolism has been increasingly reported. The aim of this systematic review was to explore the incidence of venous and arterial thromboembolism among COVID-19 patients requiring hospitalization.

**Methods:**

Medline, Embase, Scopus, and grey literature were searched until June 2020. Observational studies reported on the incidence of venous thromboembolism (VTE), including pulmonary embolism (PE) and deep vein thrombosis (DVT) or arterial thromboembolism (ATE) were included. The pool incidences and their 95% confidence intervals (CI) were calculated using the random-effects model.

**Results:**

A total of 36 studies were included. In the intensive care unit (ICU) setting, the pooled incidence of VTE was 28% (95% CI, 22–34%). Subgroups based on compression ultrasound (CUS) screening revealed a higher incidence of DVT in the CUS screening group than in the no CUS screening group (32% [95% CI, 18–45%] vs. 6% [95% CI, 4–9%]). The pooled incidence of ATE in ICU was 3% (95% CI, 2–5%). In the non-ICU setting, the pooled incidence of VTE was 10% (95% CI, 6–14%,).

**Conclusions:**

The incidence of VTE in COVID-19 patients was higher in the ICU setting than in the non-ICU setting, and also significantly higher in studies that incorporated the CUS screening protocol. The incidence of ATE in the ICU setting was low. VTE prophylactic measures should be given to all hospitalized patients diagnosed with COVID-19.

**Supplementary Information:**

The online version contains supplementary material available at 10.1186/s12959-020-00248-5.

## Background

Since December 2019, coronavirus disease 2019 (COVID-19) has emerged as a pandemic, causing high morbidity and mortality. The association between coagulation abnormalities, including disseminated intravascular coagulation and hypercoagulable state, and COVID-19 has been increasingly reported. The proposed underlying mechanism is that coronavirus infection could activate multiple systemic coagulation and inflammatory responses. Host inflammatory responses result in increased proinflammatory cytokine production, which leads to activation of coagulation and consumptive coagulopathy [1]. Several observational studies demonstrated a higher incidence of venous thrombotic events in patients diagnosed with COVID-19 admitted to the intensive care unit (ICU) compared with those from historical data [2, 3]. For arterial thrombosis, sepsis-induced coagulopathy with vascular endothelial dysfunction could contribute to microcirculatory changes in those diagnosed with COVID-19. However, few studies have reported on arterial thrombotic events in patients with COVID-19 [4]. In addition, most studies were case series and case report, which preclude the estimate of the incidence [5–7]. Anticoagulant prophylaxis is recommended by expert consensus for all critically ill COVID-19 patients, although breakthrough venous thrombosis was reported [8–10].

To date, no pre-existing systematic review and meta-analysis has addressed this issue. We conducted this systematic review to demonstrate the pooled incidence of venous and arterial thromboembolism in COVID-19 patients in various settings. The protocol was registered in PROSPERO (ID CRD42020182981).

## Methods

### Data source and literature search

A literature search was performed through bibliographic databases, including MEDLINE/Pubmed (1946 to present) using the OVID platform, Embase, and Scopus. Grey literature was searched through Google scholar and pre-print servers, including MedRxiv and SSRN. For MEDLINE and Embase, search terms were available in the supplementary index. Lists of references of relevant articles and reviews were manually reviewed and screened for potential eligibility. There was no language restriction and no filtered used for study design. Studies with languages other than English were translated using the Google Translate tool. The search was performed on May 7th, 2020. We updated the search in grey literature on June 30th, 2020.

### Study selection

Two researchers (K.B and P.C) independently screened titles and abstracts of the retrieved studies using inclusion and exclusion criteria. Review articles and references were searched for the possible included studies. Studies met eligibility criteria were included. Studies were eligible if the study design was an observational study that reported the incidence or prevalence of venous or arterial thrombosis in patients with confirmed COVID-19 requiring hospitalization. Studies with data available for incidence calculation were also included. Studies that were case-series that did not have data available for incidence calculation were excluded. Studies that were case-report, review, comments, consensus, or guidance in design were excluded. The outcome of venous thromboembolism (VTE) included pulmonary embolism (PE) and deep vein thrombosis (DVT) which were proximal, distal, and catheter-related DVT. The outcome of arterial thrombosis included ischemic stroke, myocardial infarction, and limb ischemia.

Full-text eligibility was assessed by two independent researchers (K.B and P.C). The disagreement was solved through discussion between the two researchers. If the disagreement persisted, the decision was made by the third adjudicator (A.P).

### Data abstraction and quality assessment

Two independent reviewers (K.B, P.N) independently abstracted the data. Data of study (year, author, study design), patients characteristics (age, sex, comorbidity), the severity of the disease, patient’s settings including ICU or non-ICU settings, number of events in each type of thrombosis, location of PE, number of patients requiring hospitalization, ICU admission, or non-ICU admission were recorded. If available, the number of fatal PE, thromboembolic-related mortality, and all-cause mortality were also recorded. Upon data abstraction, we found variable inclusion criteria in each study. We have categorized the included studies in clinical and imaging studies. Clinical studies were studies that recorded the incidence of thrombotic events based on clinical data. Imaging studies were studies that recorded the incidence of thrombotic events based on imaging.

The risk of bias was assessed by two independent researchers (K.B, P.N). The disagreement was solved through discussion between two researchers. Regarding no standard of risk of the bias assessment tool [11], we used the risk of bias in the prevalence study proposed by Hoy et al. [12], which comprised four domains for external validity and six domains for internal validity. Criteria for external validity comprised representation of the target population, random selection, minimization of non-response bias. Criteria for internal validity comprised data collection, acceptable definition of the outcome, reliability, and validity in measurement tool, length of follow-up, and the correction of incidence report. We acknowledged that this assessment tool was aimed for a population-based prevalence study. Thus some criteria might not be applicable to our included studies.

## Statistical analysis

The baseline characteristics of each study were summarized. Pooled incidences and respective confidence intervals (CI) of venous and arterial thrombosis were calculated from proportions extracted from reported studies. Clinical study and imaging study were analyzed separately. Meta-analysis was performed using an exact binomial random-effects model with inverse variance weighting method as we expected high heterogeneity between patients’ populations, various diagnostic utilities, and preventive strategies. Continuity correction were applied on calculations made on studies which reported zero patients. We prespecified subgroup analyses based on clinical severity, eastern and western countries, and the use of anticoagulant prophylaxis. However, most clinical studies reported outcomes only in the ICU setting with only few reported outcomes in both ICU and/or non-ICU settings. Therefore, we did not perform a subgroup analysis based on clinical severity. Furthermore, we found that some studies had a protocol for leg compression ultrasound (CUS) screening. We added a post hoc subgroup analysis of studies with leg CUS screening since these could lead to increased DVT incidence. We also performed and reported subgroup analysis based on country of studies to illustrate the differences in the incidence of VTE. Heterogeneity was explored using the Cochrane Q test. A *p*-value of < 0.05 was considered statistically significant. I^2^ statistic was calculated to estimate heterogeneity. All statistical analyses and meta-analyses were performed using the metaprop command [13] with Stata software version 16 (Stata, College Station, TX, USA).

## Results

Search results and flow of search strategies are available in the supplementary index. We identified a total of 762 articles (395 articles from Medline, 320 articles from Embase, 47 from Scopus). Grey literature search revealed 1900 articles from Google scholar, MedRxiv, and SSRN. After duplicates were removed, there were 1126 articles among which we underwent title and abstract screening. Of these, 935 articles were excluded. The remaining 191 full-text articles were assessed for eligibility criteria, and 170 articles were excluded (Supplementary index). The most common reasons for exclusion were case report or case series, which did not provide data or incidence reports and review articles. A total of 22 studies were included in the analysis. We performed a grey literature search on June 30th, 2020, and found 14 articles relevant and met the eligibility criteria.

### Risk of bias assessment

All 36 studies were assessed for risk of bias using risk of bias for prevalence studies [12]. Most studies were subjected to moderate risk of bias owing to the representative of the population since they specifically reported outcomes in ICU or non-ICU settings for which were not representative of all hospitalization with COVID-19. All but seven studies were retrospective in design. All imaging studies have a high risk of bias due to selective patients who underwent imaging studies. In addition, the indications for computed tomography pulmonary angiography (CTPA) or CUS were varied between studies. We found that the criterion on national representative of the population was not applicable to the included studies since they were not population-based prevalence studies. The internal validity criterion of prevalence period was also not applicable due to, in our study, we intended to assess the prevalence of symptoms/complications (thrombotic outcomes) rather than the prevalence of the actual disease. The risk of bias assessment is presented in supplementary index.

### Characteristics of the included studies

A total of 36 studies were included. Characteristics of the included studies are shown in Tables 1 and 2. Twenty-nine studies were retrospective, and 7 studies were prospective studies. Twenty-seven studies were from Europe (9 from France, 6 from Italy, 3 from the Netherland, 4 from Spain, 2 from the United Kingdom (UK), one each from Belgium, Germany and Switzerland). Three studies were from the United States of America (USA). Six studies were from China. There were 28 clinical and 8 imaging studies. The diagnosis of COVID-19 in most studies required the detection of severe acute respiratory syndrome coronavirus 2 (SARS-CoV-2) by real-time polymerase chain reaction (RT-PCR), but some were based on high clinical suspicion without the PCR results. In the clinical studies, 16 and 5 studies reported the incidences of outcomes only in the ICU [2, 3, 8, 14–26] or non-ICU settings [27–31], respectively. Seven studies reported the incidence of outcomes in the ICU, and non-ICU settings [4, 9, 10, 32–35]. Six studies reported on both venous and arterial thrombosis [2, 14, 16, 18, 21, 33]. Twenty-nine studies reported only venous thrombotic events, and one study reported only arterial thrombotic events [4]. Among 23 in 29 studies reported venous thrombotic outcomes, utilized anticoagulant prophylaxis. In the imaging studies, 6 studies mainly focused on CTPA in all hospitalization patients [36–41], and 2 studies focused on using compression ultrasound (CUS) in a non-ICU setting [42, 43].

**Table 1:** Characteristics of the clinical studies

Author	Study design	Country	Patient population	VTE Events/Total patients (%)	Age, mean (SD)^a^	Male sex,% ^a^	Anticoagulant prophylaxis	Indication for CTPA	CUS screening
Beun [14]	Retrospective cohort	Netherlands	ICU	23/75 (30.7)	VTE: 60.5 (min-max, 53–68)	NR	NR	NR	NR
Cui [15]	Retrospective cohort	China	ICU	20/81 (24.7)	VTE: 68.4 (9.1) Non-VTE: 57.1 (14.3)	46	No	CT, assumed in all patients	Yes
Desborough [24]	Retrospective Cohort	UK	ICU	11/79 (13.9)	VTE: 54 (45–63) No VTE: 59 (52–67)	73	Yes	Clinical suspicion	No
Fraissé [21]	Retrospective cohort	France	ICU	31/92 (33.7)	61 (55–70)^b^	79	Yes	Clinical suspicion	No
Helms [2]	Prospective cohort	France	ICU	28/150 (18.7)	63 (53–71) ^b^	81.3	70% PD, 30% TD	Clinical suspicion or rapid D-dimer elevation	NR
Hippensteel [26]	Retrospective cohort	USA	ICU	24/107 (22.4)	VTE: 55 (13) No VTE: 57 (17)	VTE: 14 No VTE: 39	NR	Clinical suspicion	No
Klok [16, 44]	Retrospective cohort	Netherlands	ICU	68/184 (37)	64 (12)	76	Yes, adjust per BW 9% TD	Clinical suspicion	No
Llitjos [8]	Retrospective cohort	France	ICU	20/26 (76.9)	68 (51.5–74.5) ^b^	77	31% PD, 69% TD	Clinical suspicion	Yes, 1st CDU on day 1–3 and 2nd CDU on day 7
Longchamp [19]	Prospective cohort	Switzerland	ICU	8/25 (32)	68 (11)	64	Yes	Clinical suspicion	Yes, D5-D10
Nahum [23]	Prospective cohort	France	ICU	27/34 (65)	62.2 (8.6)	78	Yes	NR	Yes
Poissy [3]	Retrospective cohort	France	ICU	27/107 (25.2)	PE: 57 (29–80) ^b^	PE: 59.1	91% PD, 9% TD	Clinical suspicion	Partially performed
Soumagne [22]	Prospective cohort	France and Belgium	ICU	79/375 (21)	63.5 (10.1)	77	NR	NR	NR
Spiezia [20]	Prospective cohort	Italy	ICU	5/30 (16.7)	VTE: 67 (8)^d^	90^d^	Yes	NR	NR
Zerwes [25]	Prospective cohort	Germany	ICU	4/40 (10)	63.4 (18.1)	67.5	Yes	NR	Yes
Thomas [18]	Retrospective cohort	UK	ICU	6/63 (9.5)	59 (13)	69	Yes, adjust per BW	Clinical suspicion	NR
Tavazzi [17]	Retrospective cohort	Italy	ICU	10/54 (18.5)	VTE: 68 (7)	VTE: 83	Yes, adjust per BW	NR	No
Demelo-Rodríguez [27]	Prospective cohort	Spain	Non-ICU	23/198 (11.6) CUS done in 156	DVT: 66.7 (15.2) No DVT: 68.4 (14.4)	DVT: 60.9 No DVT: 66.2	Yes, 98%	NR	Yes, d-dimer > 1000 & hospitalization > 48 h
Dubois-Silva [29]	Retrospective cohort	Spain	Non-ICU	8/171 (4.9)	PE: 67 (58–74)^b^	62.5	Yes	Clinical suspicion	Yes
Mazzaccaro [31]	Retrospective cohort	Italy	Non-ICU	21/32 (65.6)	68.6 (12)	71.9	Yes	All patients	Yes
Mestre-Gómez [30]	Retrospective cohort	Spain	Non-ICU	31/452 (6.9)	PE: 65 (56–73)^b^	72	Yes, partial	Clinical suspicion	No
Zhang [28]	Retrospective cohort	China	Non-ICU	67/159 (42.1) CUS done in143	DVT: 67 (12) No DVT: 59 (16)	51.7 DVT: 54.5 No DVT: 49.4	Yes, 37%	Clinical suspicion	Yes
Criel [34]	Prospective cohort	Belgium	Inpatients	Total: 82 ICU: 4/30 (13.3), Ward: 2/52 (3.8)	ICU: 64.5 (11.8) Non-ICU: 63.6 (14.4)	ICU: 67 Non-ICU: 54	Yes, adjust per BW	Not done	Yes
Koleilat [35]	Retrospective case-control	USA	Inpatients	93/3403 (2.7) CUS done in 846	DVT:59 (49–64) No DVT 64 (53–73)	DVT: 61.1 No DVT:61 52.1	Yes, partial	NR	No
Logigiani [33]	Retrospective cohort	Italy	Inpatients	Total: 388 ICU: 8/61 (13.1) Ward: 12/327 (3.7)	ICU: 61 (55–69)^b^ Ward: 68 (55–77)	68.0 ICU: 80.3 Ward: 65.7	ICU: 100%, Ward: 75% 41% PD, 21% ID, 23% TD	Clinical suspicion or rapid increase in d-dimer	No
Middeldorp [9]	Retrospective cohort	Netherlands	Inpatients	Total:198 ICU: 39/75 (52) Ward: 4/123 (3.3)	ICU: 62 (10) Ward: 60 (16)	66 ICU: 77 Ward: 59	Yes, adjust per BW 84% PD 9.6% TD	Clinical suspicion Sudden worsening hypoxemia	Yes, partial 28% of all
Mao^e^ [4]	Retrospective cohort	China	Inpatients	Total: 214 Severe: 88 Non-severe: 126	Severe: 58.2 (15.0) Non severe: 48.9 (14.7)	40.7 Severe: 50 Non severe: 34.1	NR	NR	NR
Wang^c^ [32]	Retrospective cohort	China	Inpatients	Total: 88 Critical+severe: 20/63 (31.7) Common: 0/25	Critical: 66.5 (61–71)^b^ Severe: 61.0 (53–66) Common: 56 (42.5–66.5)	55.7 Critical: 70 Severe: 42.4 Common: 56	Yes, according to Padua risk score	Clinical suspicion	Yes, increased d-dimer
Xu^c^ [10]	Retrospective cohort	China	Inpatients	Total: 138 Critical+severe: 3/15 (20) Non-critical: 1/123 (0.8)	Critical: 60.07 (14.3) Non-critical: 50.5 (16)	58.7 Critical: 80 Non-critical: 56	Yes Critical 100% Non-critical 21.5%	In those performed CUS	Yes, all critically ill, high risk of VTE, high level d-dimer

^a^ All patients included in the study, ^b^ Median (IQR), ^c^ categorized patients on clinical severity, ^d^ of those 22 patients met the inclusion criteria of the study, ^e^ reported only arterial events, and CT Brain was performed according to clinical needs, *BW* Body weight, *NR* Not reported, *ICU* Intensive care unit, *VTE* Venous thromboembolism, *PD* Prophylactic dose, *IT* Intermediate dose, *TD* Therapeutic dose

**Table 2:** Characteristics of the imaging studies

Author	Study design	Country	Population	Type of Imaging	Total imaging performed	Anticoagulant prophylaxis	Indication for imaging	CUS screening
Bompard [36]	Retrospective cohort	France	All underwent CTPA	CTPA	135 PE: 32 No PE: 103	Yes	Clinical suspicion	NR
Chen [37]	Retrospective cohort	China	Inpatients underwent CTPA	CTPA	25 PE: 10 No PE: 15	NR	Clinical suspicion	No
Franco-Lopez [38]	Retrospective cohort	Spain	Inpatients underwent CTPA	Angio CTs	18	NR	Increase in D-Dimer	No
Grillet [39]	Retrospective cohort	France	Inpatients underwent CTPA	CT scan	All 100 PE: 23 No PE: 77	NR	All severe cases	No
Leonard-Lorant [40]	Retrospective cohort	France	All underwent CT	CTPA	106 PE: 32 No PE: 74	Yes, 46%	Clinical suspicion 63% Others 37%	No
Poyiadji [41]	Retrospective cohort	USA	All underwent CTPA	CTPA	328 PE:72 No PE: 256	Yes, partial	NR	NR
Marone [43]	Retrospective cohort	Italy	Inpatients underwent CUS Non-ICU	CUS	30	No	Clinical suspicion	Clinical suspicion
Cattaneo [42]	Retrospective cohort	Italy	Inpatients underwent CUS Non-ICU	CUS	64	Yes	Screening	Yes, asymptomatic

*NR* Not reported, *ICU* Intensive care unit, *VTE* Venous thromboembolism, *PE* Pulmonary embolism, *DVT* Deep vein thrombosis, *CTPA* Computed tomography pulmonary angiography, *CUS* Compression ultrasonography

#### Clinical study

##### VTE in ICU setting

**Fig. 1:**
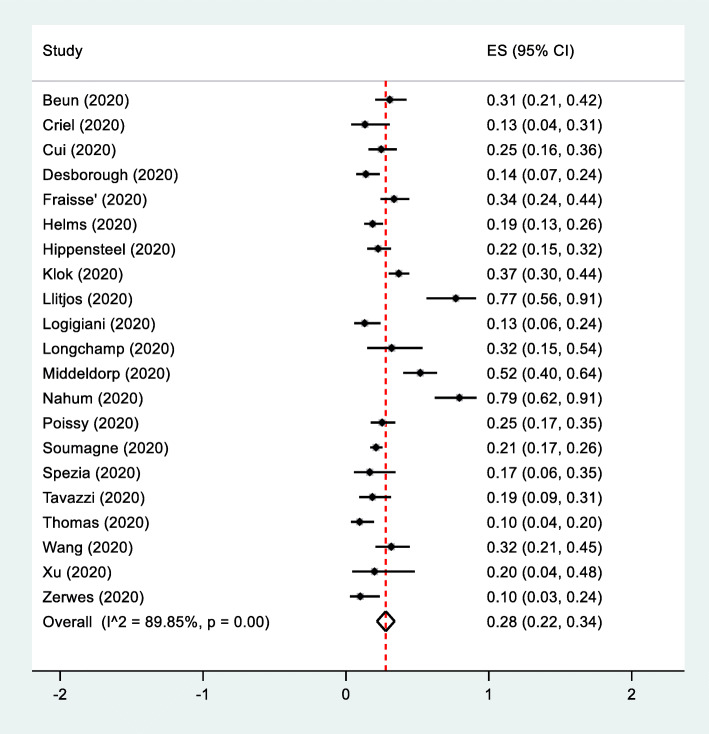
The pooled incidence of VTE from clinical studies in the ICU stteing. Forest plot of studies showed the pooled incidence of VTE from clinical studies in the ICU setting. The analysis included 21 clinical studies. VTE occurred in 465 of 1766 patients with COVID-19 admitted in the ICU. *P* value for heterogeneity was less than 0.001

A total of 21 clinical studies were included. VTE occurred in 465 of 1766 patients with COVID-19 admitted in the ICU. The pooled incidence of total VTE, including DVT and PE was 28% (95% CI, 22–34%, I^2^ = 89.5) (Fig. 1). The pooled incidence of PE was 3% (95% CI, 2–4%, I^2^ = 94.3). The pooled incidence of DVT was 15% (95% CI, 11–20%, I^2^ = 92.6) (Figures S1 and S2 in the supplementary index).

**Fig. 2:**
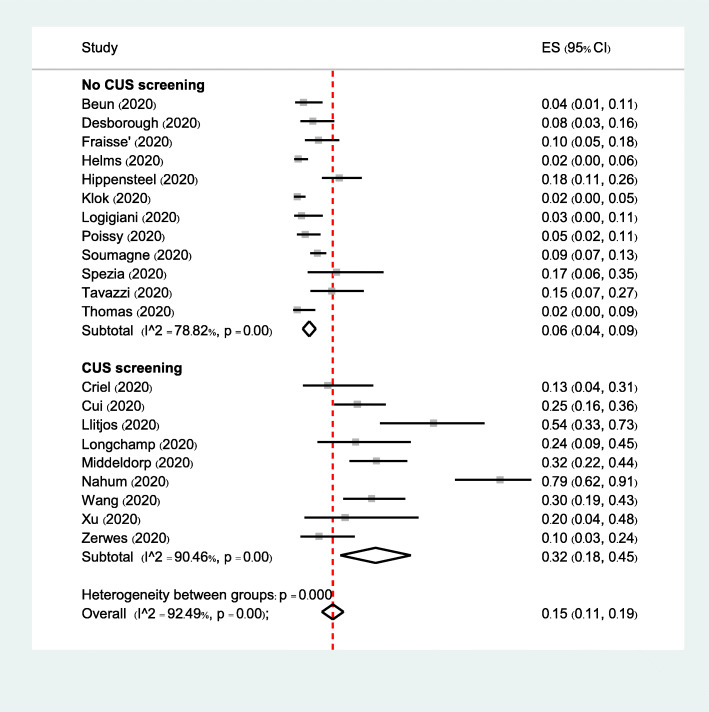
Subgroup analysis of the pooled incidence of DVT from clinical studies in the ICU setting based on CUS screening. Forest plot of subgroup analysis of the incidence of DVT in the ICU setting based on studies with CUS screening. There were 12 studies with no CUS screening and 9 studies with CUS screening. DVT were found in 102 of 1377 patients with no CUS screening and in 121 of 389 patients with CUS screening. *P* value for heterogeneity between groups was less than 0.001

Subgroup analyses of VTE based on anticoagulant prophylaxis, eastern or western countries, and CUS screening did not reveal significant differences between subgroups (Figures S3-S5 in the supplementary index). When focusing on the incidence of DVT, subgroup analysis based on CUS screening demonstrated a significant interaction (*p* < 0.001). In 12 studies with no CUS screening, the incidence of DVT was 6% (95% CI, 4–9%), whereas in 9 studies with CUS screening, the incidence of DVT was 32% (95% CI, 18–45%) (Fig. 2).

**Fig. 3:**
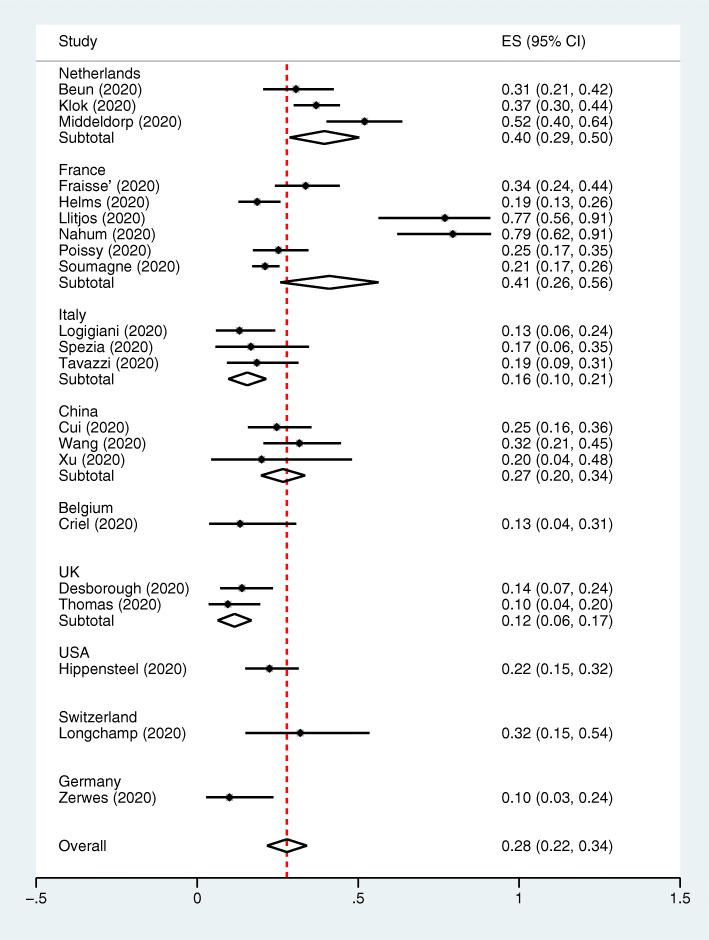
The pooled incidence of VTE from clinical studies in the ICU setting by country. Forest plot of the subgroup analysis of the incidence of VTE in the ICU setting based on countries. Three studies were from the Netherlands where VTE occurred in 130 of 334 patients. Six studies from France where VTE occurred in 212 of 784 patients. Three studies from Italy where VTE occurred in 23 of 145 patients. Three studies from China where VTE occurred in 43 of 162 patients. One study from Belgium where VTE occurred in 4 of 30 patients. Two studies from UK where VTE occurred in 17 of 142 patients. One study from Switzerland where VTE occurred in 8 of 25 patients . One study from USA where VTE occurred in 24 of 107 patients. One study from Germany where VTE occurred in 4 of 40 patients

Overall, studies from the Netherlands, France, China, Italy and UK demonstrated the pooled VTE incidence of 40% (95% CI, 29–50%), 41% (95% CI, 26–56%), 27% (95% CI, 20–34%), 16% (95% CI, 10–21%), and 12% (95% CI, 6–17%), respectively. Each study from Switzerland, USA, Belgium and Germany demonstrated the VTE incidence of 32% (95% CI, 15–54%), 22% (95% CI, 15–32%), 13% (95% CI, 4–31%), and 10% (95% CI 3–24%), respectively (Fig. 3).

##### VTE in non-ICU setting

A total of 10 clinical studies reported VTE events in non-ICU setting were included in the analysis. In 171 of 1662 patients with COVID-19 admitted in the general ward, the pooled incidence of total VTE was 10% (95% CI, 6–14%, I^2^ = 96.8). The pooled incidence of PE was 0%. The pooled incidence of DVT was 1% (95% CI, 1–2%, I^2^ = 96) (Figures S6-S8 in the supplementary index).

Subgroup analysis of VTE based on anticoagulant prophylaxis was not performed since all had anticoagulant prophylaxis. There was no significant interaction on subgroup analysis based on eastern or western countries. Subgroup analyses based on CUS screening revealed a significant interaction between subgroups (*p* = 0.007). In 8 studies of the CUS screening subgroup, the incidence of VTE was 12% (95% CI, 7–17%). In 2 studies of the no CUS screening subgroup, the incidence of VTE was 5% (95% CI, 2–6%) (Figure S9-S10 in supplementary index).

##### Arterial thrombosis in the ICU setting

A total of 7 clinical studies in the ICU setting reported on arterial thrombotic events, including myocardial infarction, ischemic stroke, and limb ischemia. Arterial thrombosis occurred in 30 of 713 patients with COVID-19 admitted in the ICU. The pooled incidence of total arterial thrombosis was 3% (95% CI, 2–5%, I^2^ = 4.1) (Figure S11 in the supplementary index).

##### Arterial thrombosis in the non-ICU setting

Two clinical studies reported on arterial thrombotic events including ischemic stroke and myocardial infarction in the non-ICU setting. Arterial thrombosis occurred in 10 of 453 patients with COVID-19 admitted in the non-ICU. The pooled incidence of total arterial thrombosis was 2% (95%CI, 0–3%, I^2^ = 0) (Figure S12 in the supplementary index).

##### Mortality

Six clinical studies reported the number of patients with VTE who died in the ICU setting. The overall mortality rate was 6% (3–10%, I^2^ = 63.8) (Figure S13 in the supplementary index).

#### Imaging studies

A total of 8 imaging studies were included. VTE was found in 261 of 949 imaging performed in patients with COVID-19 requiring hospitalization. The pooled incidence of total VTE was 29% (95% CI, 15–42%, I^2^ = 97.5) (Figure S14 in the supplementary index). Since each imaging study focused and reported on a specific type of imaging, we analyzed separately for imaging studies focusing on either CTPA or CUS study. In six imaging studies focusing on CTPA, the pooled incidence of PE was 26% (95% CI, 21–31%, I^2^ = 40.8). In 2 imaging studies focusing on CUS, the pooled incidence of DVT was 0% (Figure S15-16 in supplementary index).

For the location of PE reported on imaging studies focusing on CTPA, one study did not report the location of thrombus, and one study reported sites of thrombus of subsegmental, segmental, and lobar artery together. Four imaging studies focusing on CTPA reported the location of thrombus by distal (subsegmental, segmental artery) vs. proximal (lobar and more proximal part) artery. Of 144 PE detected, distal PE was found in 81 (56%), and proximal PE was found in 36 (35%).

## Discussion

Since evidence of an increased risk of thromboembolism in hospitalized COVID-19 patients emerged, several observational studies have reported specific outcomes on venous or arterial thrombosis. We systematically searched and illustrated the pooled incidence of venous and arterial thromboembolism in various clinical settings. We found that in patients requiring ICU admission had a higher incidence of VTE (28%) than those in a non-ICU setting (10%). Several studies demonstrated a significant increase in D-dimer and fibrinogen levels, which reflected the hypercoagulability state in COVID-19 ICU patients [20, 45]. It is hypothesized that in severe clinical COVID-19 pneumonia, the massive release of inflammatory mediators caused by viral replication might be contributed to endothelial injury and intravascular thrombosis [46]. Our findings support the association between clinical severity and hypercoagulable state causing by COVID-19.

In the ICU setting, the pooled incidence of VTE was 28% with high heterogeneity. Prespecified subgroup analyses, including anticoagulant prophylaxis, provided similar results as the primary analysis. The interpretation of this subgroup analysis requires caution since only 4 studies did not utilize anticoagulant prophylaxis. In addition, among studies that utilize anticoagulant prophylaxis, criteria for anticoagulant prophylaxis and the dosage are varied. However, our data demonstrated that breakthrough VTE on anticoagulant prophylaxis occurred in approximately 29% in the ICU setting. In patients with COVID-19 with severe clinical severity or required ICU admission, prophylactic-intensity anticoagulation might not be sufficient. Whether a higher intensity anticoagulant could effectively prevent the venous thrombotic events in critically ill patients with COVID-19 is unknown. Several randomized controlled trials looking at the appropriate intensity of LMWH prophylaxis are still ongoing.

Our finding of a high incidence of VTE in the ICU setting was likely driven by the incidence of DVT rather than PE. The pooled incidence of PE was lower than we expected. When we performed post hoc subgroup analysis based on countries, studies from the Netherland and France had a higher pooled incidence of PE ranged from 17 to 27%, whereas the studies from Italy and UK reported the lower incidence of PE, which ranged from 3 to 7%. The significant between-country differences in incidence reflect more stringent diagnostic workup procedures in the countries with higher reported incidence. Other confounding factors also could contribute to this finding. The indications for CTPA were varied between studies. Most studies performed CTPA based on clinical suspicion, whereas some studies based on a high D-dimer level or only in patients with DVT. Several studies did not mention performing CTPA or the indication for CTPA, which could underestimate the incidence of PE.

In China, the incidence of VTE in the ICU setting was 26%. Here in our academic center in Thailand, we also found a significant number of PE in severe COVID-19 pneumonia patients. There were 3 symptomatic PE out of 14 severe COVID-19 pneumonia requiring ICU admission. At the time of events, all three patients did not receive anticoagulant prophylaxis [47]. Therefore, the incidence of VTE in severe COVID-19 requiring ICU admission in our center was 21.4%. There was no VTE among 130 non-severe COVID-19. It is noted that we did not perform routine CUS screening in our patients.

The incidence of DVT in the ICU setting was significantly higher in studies that performed CUS screening than those studies which did not (32% vs. 6%, respectively). Though this finding was as expected since the more imaging performed, the more DVT events detected. The significance of asymptomatic DVT in the ICU setting detected by CUS is still debating. Whether a high incidence of DVT detected on CUS has an impact on the development of PE or mortality was unknown. Given the high risk of transmission of SARS-CoV-2 to health care personnel, recent expert guidance suggests against routine CUS screening in patients with COVID-19 requiring ICU admission [48].

The incidence of VTE in COVID-19 in the non-ICU setting was 10%. Subgroup analysis based on CUS screening revealed significant interaction. However, the credibility of the subgroup analysis was low. Most VTE incidences were driven by DVT dominating by Zhang et al. study (DVT incidence =42%) and by PE dominating by Mazzaccaro et al. study (PE incidence = 72%, respectively) [28, 31]. In Zhang study, anticoagulant prophylaxis was given in 37% of patients whereas in other studies anticoagulant prophylaxis was utilized in more than 80% of patients. In another study from China by Xu and coworkers [10], anticoagulant prophylaxis was also partially given in 22% of patients, but the incidence of DVT was quite low (1%). This could be explained by a difference in the baseline risk of VTE for which the proportion of patients with high-risk VTE (Padua score ≥ 4) was much higher in the study by Zhang than those in the study by Xu et al. (65% vs. 7%). A high incidence of PE in a study by Mazzaccaro could be due to CTPA screening for PE in all patients rather than in those with clinical suspicion. Overall, the incidence of VTE is low in the non-ICU setting. Anticoagulant prophylaxis should be considered in the non-ICU setting, especially in patients with high risk for VTE.

Among the imaging studies which reported PE events detected by CTPA or DVT detected by CUS, the incidences of PE and DVT were 26 and 33%, respectively. Most studies selected patients who underwent CTPA or CUS regardless of clinical severity status. Thus, the interpretation was limited. Findings of imaging on CTPA demonstrated that thrombus was more commonly occurred in the distal part (subsegmental and segmental artery) rather than a more proximal pulmonary artery. This could reflect the microvascular thrombosis “in situ” caused by endothelial injury and local inflammation [46, 49].

Data on VTE-related death were limited. Few studies reported the outcome of death in patients with VTE, but it could not be assumed to be VTE-related death in all patients. Many clinical and laboratory factors, including older age, sex, clinical severity assessed by using SOFA score, and high D-dimer were associated with mortality [50, 51].

Few arterial thrombotic events have been reported. Most events were ischemic stroke, and few were myocardial infarction. Though several case reports and case series demonstrated the possibly increased risk of arterial thrombosis, we did not include those studies in the analysis since there was no data available for incidence calculation, and the study designs are subjected to selection bias.

Our study has several strength. We performed a systematic literature search, including grey literature with no language restriction. Full-text eligibility and risk of bias were reviewed by two independent researchers. However, there are some limitations. Most studies were retrospective in design with a small sample size. Thus they were subjected to risk of selection bias. High heterogeneity between groups was presented in most analyses, although I^2^ is possibly not a reliable indicator of true heterogeneity among prevalence meta-analyses [52]. One post hoc subgroup analysis was able to demonstrate the subgroup effect but prespecified subgroup analyses did not reveal significant interaction. CTPA for PE detection was not routinely performed in all patients in the ICU. Hence, the true incidence of PE could be underestimated. However, most studies performed CTPA based on clinical suspicion, which represented the clinically significant PE.

## Conclusion

The incidence of VTE was 28 and 10% in COVID-19 patients in the ICU and non-ICU settings, respectively. The incidence of DVT was significantly higher in studies that incorporated the CUS screening protocol. The incidence of ATE in the ICU setting was low. VTE prophylactic measures should be given to all hospitalized patients with COVID-19, especially in the ICU setting. Since approximately one-fourth of patients admitted to the ICU setting developed VTE, careful monitoring of the patients for VTE and its complications is strongly advised. The optimization of anticoagulant dosing to prevent VTE is currently under investigation.

## Supplementary Information

**Additional file 1.**

## Data Availability

Not applicable.

## Abbreviations

COVID-19: Coronavirus disease 2019
VTE: Venous thromboembolism
PE: Pulmonary embolism
DVT: Deep vein thrombosis
ATE: Arterial thromboembolism
CI: Confidence interval
ICU: Intensive care unit
CUS: Compression ultrasound
CTPA: Computed tomography pulmonary angiography
UK: United Kingdom
USA: United States of America
SARS-CoV-2: Severe acute respiratory syndrome coronavirus 2
RT-PCR: Real-time polymerase chain reaction
