# Correction to: Incidence of thromboembolism in patients with COVID-19: a systematic review and meta-analysis

**DOI:** 10.1186/s12959-020-00254-7

**Published:** 2020-12-29

**Authors:** Kochawan Boonyawat, Pichika Chantrathammachart, Pawin Numthavaj, Nithita Nanthatanti, Sithakom Phusanti, Angsana Phuphuakrat, Pimjai Niparuck, Pantep Angchaisuksiri

**Affiliations:** 1 Department of Medicine, Faculty of Medicine Ramathibodi Hospital, Mahidol University, Bangkok, Thailand; 2 Department of Clinical Epidemiology and Biostatistics, Faculty of Medicine Ramathibodi Hospital, Mahidol University, Bangkok, Thailand; 3 Chakri Naruebodindra Medical Institute, Faculty of Medicine Ramathibodi Hospital, Mahidol University, Samut Prakan, Thailand

**Correction to: Thromb J (2020) 18:34**

**https://doi.org/10.1186/s12959-020-00248-5**

Following the publication of the original article [1], the authors identified an error in the name of one of the authors.

The incorrect name was: Pawin Numthavej

The correct name is: Pawin Numthavaj

The author group has been updated above and the original article [1] has been corrected.
