# Supplementary material for: Incidence of thromboembolism in patients with COVID-19: a systematic review and meta-analysis
Source: Thromb J. 2020 Nov 23;18:34. doi: 10.1186/s12959-020-00248-5 (PMC7680990; doi:10.1186/s12959-020-00248-5)

Supplementary index

Medline search

Database(s): Ovid MEDLINE(R) ALL 1946 to May 06, 2020
Search Strategy: accessed 7-5-20 12.45 am

| **#** | **Searches** |
| --- | --- |
| 1 | corona.mp. |
| 2 | (virus or viruses or viral).mp. |
| 3 | 1 and 2 |
| 4 | wuhan.mp. |
| 5 | 2 and 4 |
| 6 | COVID.mp. |
| 7 | COVID19.mp. |
| 8 | COVID-19.mp. |
| 9 | nCOV*.mp. |
| 10 | SARS2.mp. |
| 11 | SARS-2.mp. |
| 12 | SARS-CoV-2.mp. |
| 13 | SARS-CoV*.mp. |
| 14 | 2019-corona*.mp. |
| 15 | 2019-nCOV*.mp. |
| 16 | 3 or 5 or 6 or 7 or 8 or 9 or 10 or 11 or 12 or 13 or 15 |
| 17 | thrombosis.mp. or Thrombosis/ |
| 18 | exp Thromboembolism/ or thromboembolism.mp. |
| 19 | venous thrombosis.mp. or exp Venous Thrombosis/ |
| 20 | venous thromboembolism.mp. or exp Venous Thromboembolism/ |
| 21 | deep vein thrombo*.mp. |
| 22 | deep venous thrombo*.mp. |
| 23 | pulmonary embolism.mp. or exp Pulmonary Embolism/ |
| 24 | pulmonary emboli*.mp. |
| 25 | lung embolism.mp. |
| 26 | lung emboli*.mp. |
| 27 | arterial thrombo*.mp. |
| 28 | exp Cerebrovascular Disorders/ or cerebrovascular dis.mp. |
| 29 | stroke.mp. or exp Stroke/ |
| 30 | (brain* or cere* or cerebell* or intracran* or intracerebral *).mp. |
| 31 | (isch?emi* or infarct* or thrombo* or emboli*).mp. |
| 32 | exp Myocardial Infarction/ or myocardial infarct*.mp. |
| 33 | heart infarct*.mp. |
| 34 | heart attack.mp. |
| 35 | 17 or 18 or 19 or 20 or 21 or 22 or 23 or 24 or 25 or 26 or 27 or 28 or 29 or 30 or 31 or 32 or 33 or 34 |
| 36 | 16 and 35 |

Embase search

No.

Query

#35

#34 AND (2019:py OR 2020:py)

#34

#15 AND #33

#33

#16 OR #17 OR #18 OR #19 OR #20 OR #21 OR #22 OR #23 OR #24 OR #25 OR #26 OR #29 OR #30 OR #31 OR #32

#32

heart AND attack

#31

'heart infarction'

#30

myocardial AND infarction

#29

#27 AND #28

#28

isch?emi* OR infarct* OR thrombo* OR emboli*

#27

brain* OR cere* OR cerebell* OR intracran* OR intracerebral*

#26

stroke

#25

'cerebrovascular accident'

#24

'cerebrovascular disease'

#23

'artery thrombosis'

#22

'lung embolism'

#21

'pulmonary embolism and thrombosis'

#20

'deep vein thrombosis'

#19

venous AND thrombosis

#18

'thromboembolism'

#17

'vein thrombosis'

#16

'thrombosis'

#15

#4 OR #5 OR #6 OR #7 OR #8 OR #9 OR #10 OR #11 OR #12 OR #13 OR #14

#14

'2019 ncov'

#13

'2019 corona'

#12

'sars coronavirus'

#11

'sars cov 2'

#10

'sars 2'

#9

sars2

#8

ncov*

#7

'covid 19'

#6

covid*

#5

#2 AND #3

#4

#1 AND #3

#3

virus OR viruses OR viral

#2

wuhan

#1

corona

Risk of bias assessment

**Flow Diagram**

Records identified through database searching
Medline (n = 395)

Embase (n= 320)

Scopus (n=47) total 762

Studies included in quantitative synthesis (meta-analysis)
(n = 36)

Records excluded
(n = 934)

Records screened
(n =1126)

Records after duplicates removed
duplicate = 389+125 =514 (n = 2299)

## Identification

## Eligibility

## Included

## Screening

Additional records identified through other sources
Google Scholar (n = 1138)

MedRxiv (n=48)

SSRN (n=2)

Full-text articles excluded, with reasons
n = 170

44 case report or series

7 comments

6 Duplicate

4 guidance

12 not relevant

32 no incidence reported

64 review

1 full text unavailable

Studies included in qualitative synthesis
(n = 36)

Full-text articles assessed for eligibility
(n =192)

| Figure S1 Forest plot of the incidences of PE from clinical studies in the ICU setting. The analysis included 20 clinical studies. PE occurred in 164 of 1009 patients with COVID-19 admitted in the ICU. P value for heterogeneity was less than 0.001. |
| --- |
| 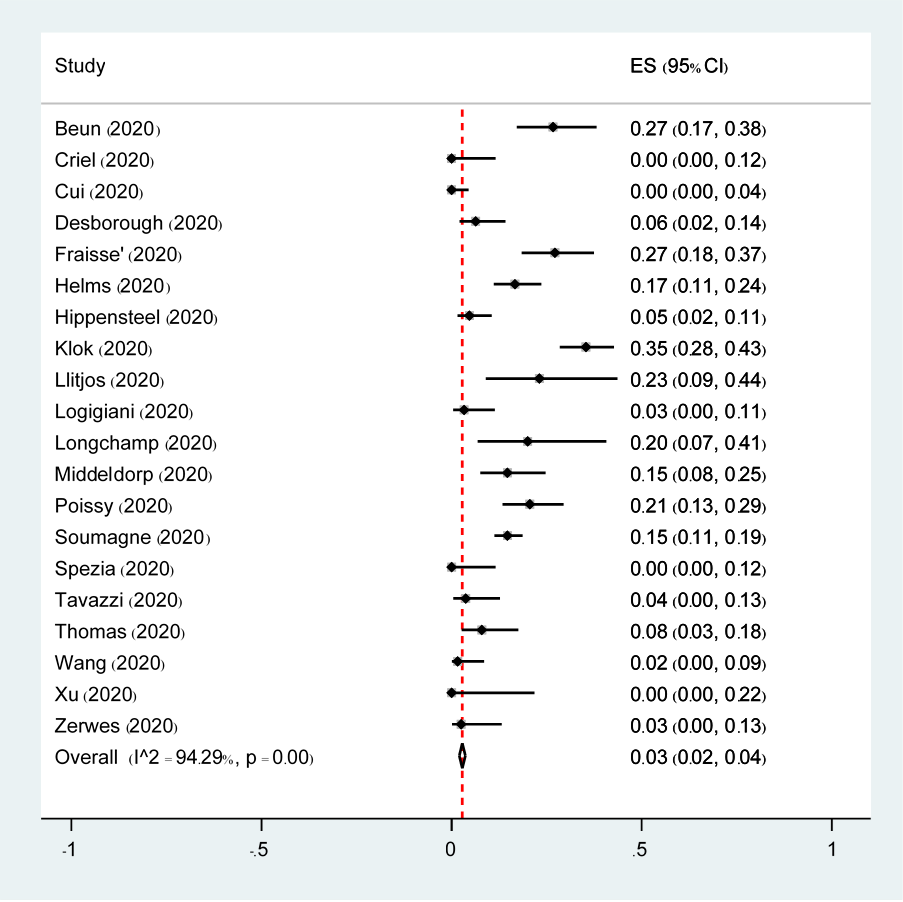 |
|  |

| Figure S2 Forest plot of the incidences of DVT from clinical studies in the ICU setting. The analysis included 21 clinical studies. DVT occurred in 120 of 1039 patients with COVID-19 admitted in the ICU. P value for heterogeneity was less than 0.001. |
| --- |
| 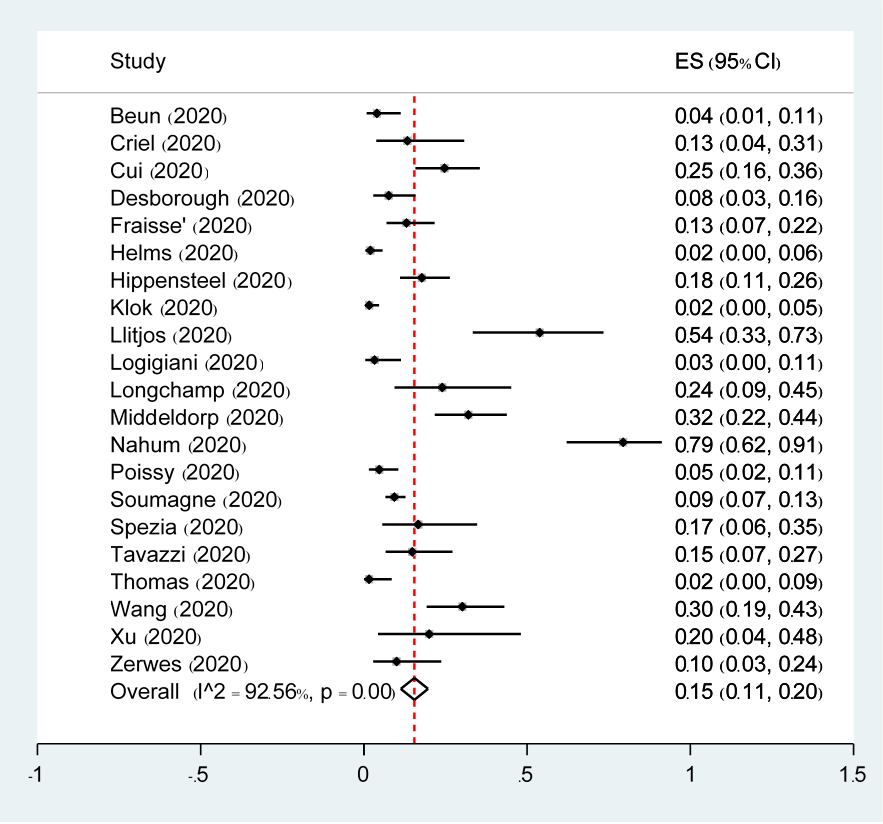 |
|  |

| Figure S3 Forest plot of subgroup analysis of the incidences of VTE in the ICU setting based on studies with AC prophylaxis. There were 4 studies with no anticoagulant prophylaxis and 17 studies with anticoagulant prophylaxis. VTE events occurred in 146 of 638 patients with no AC prophylaxis and in 319 of 1128 patients with AC prophylaxis. P value for heterogeneity between subgroups was 0.309. |
| --- |
| 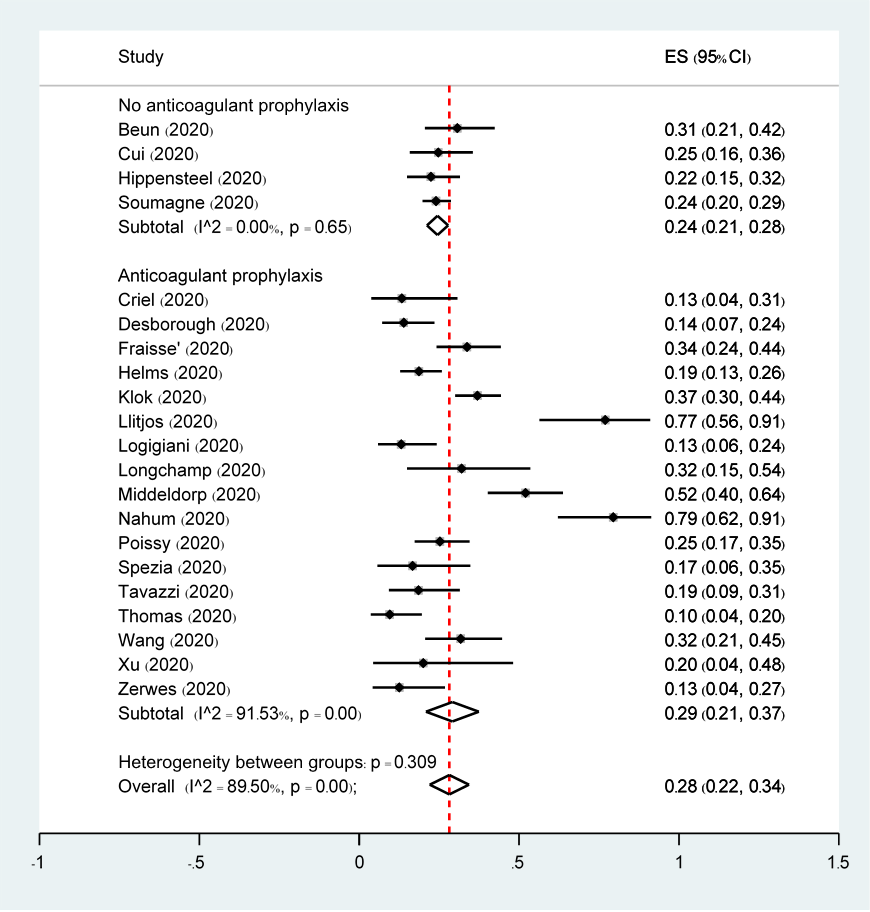 |
| \| Figure S4 Forest plot of subgroup analysis of the incidences of VTE in the ICU setting based on western or eastern countries. There were 18 studies from western and 3 from eastern countries. VTE events occurred in 422 of 1607 patients from the western coutries and in 43 of 159 patients from the eastern coutries. P value for heterogeneity between subgroups was 0.7. \| \| --- \| \|  \|   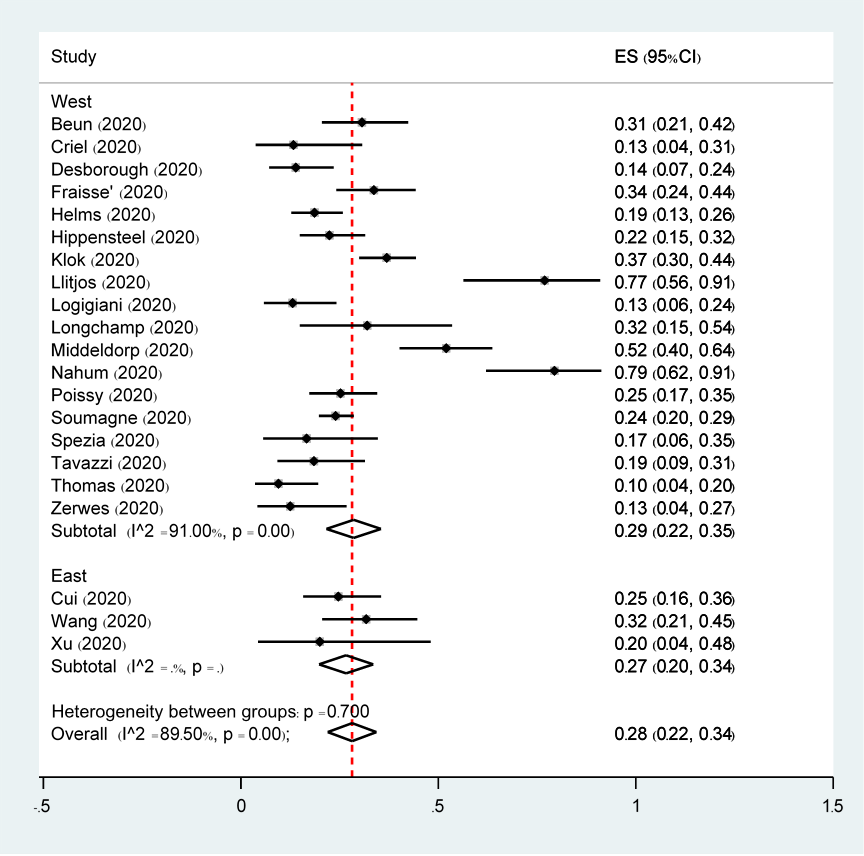 |
| Figure S5 Forest plot of subgroup analysis of the incidences of VTE in the ICU setting based on studies with CUS screening. There were 12 studies with no CUS screening and 9 studies with CUS screening. VTE were found in 320 of 1377 patients with no CUS screening and in 145 of 389 patients with CUS screening. P value for heterogeneity between subgroups was 0.06. |
| 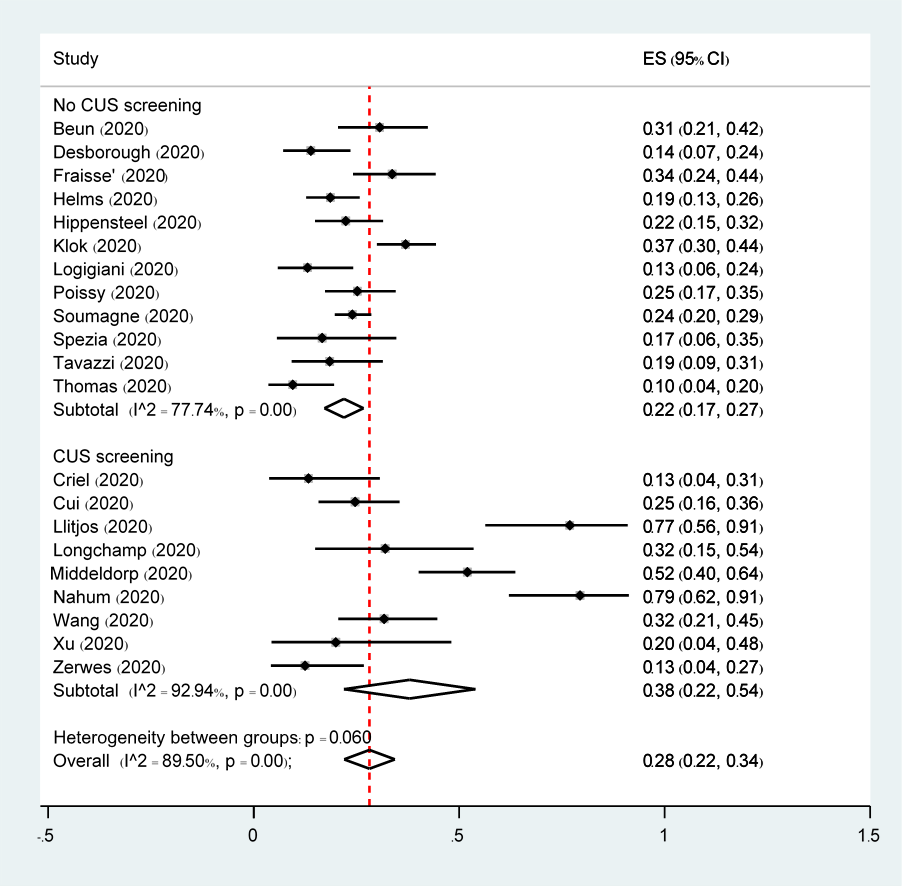 |
| Figure S6 Forest plot of the incidences of VTE from clinical studies in the non-ICU setting. The analysis included 10 clinical studies. VTE occurred in 171 of 1662 patients with COVID-19 admitted in the non-ICU. P value for heterogeneity was less than 0.001. |
| 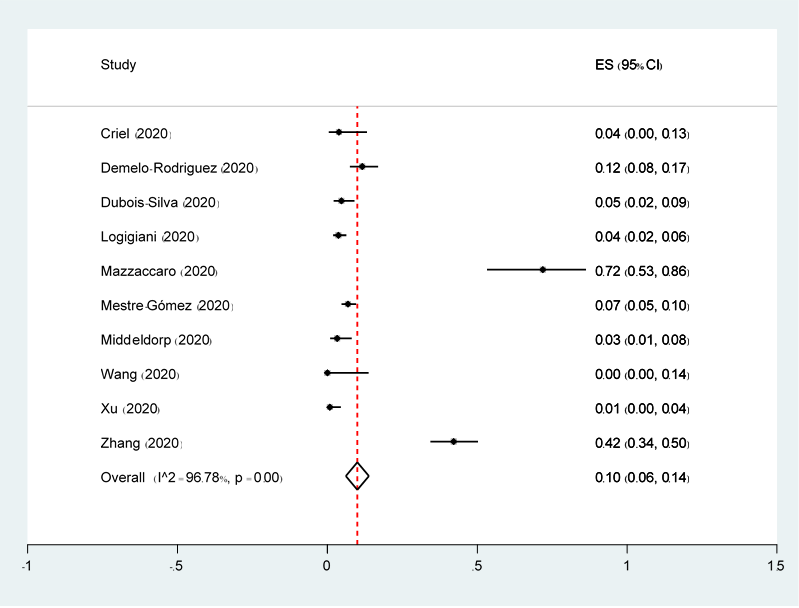 |
| Figure S7 Forest plot of the incidences of PE from clinical studies in the non-ICU setting. The analysis included 10 clinical studies. PE occurred in 69 of 1662 patients with COVID-19 admitted in the non-ICU. A continuity correction of 0.001 was utilized in studies with no event. P value for heterogeneity was less than 0.001  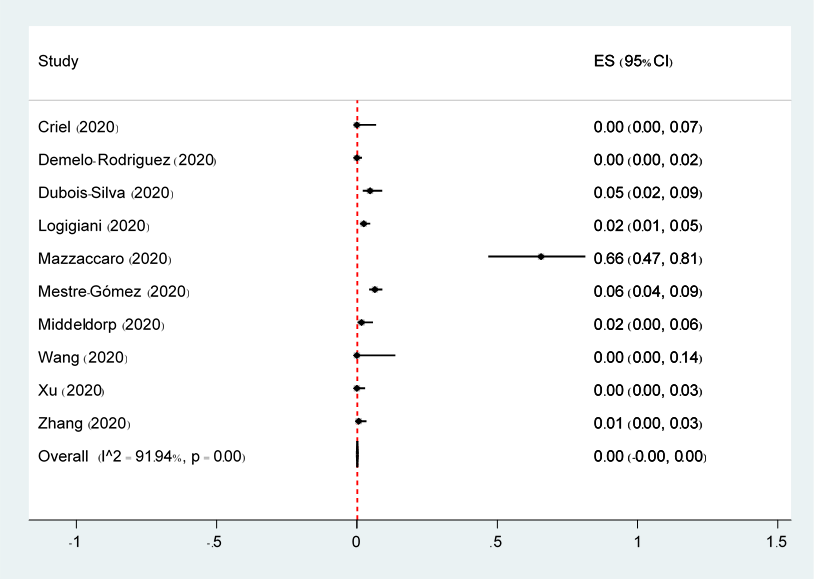 |
| Figure S8 Forest plot of the incidences of DVT from clinical studies in the non-ICU setting. The analysis included 10 clinical studies. DVToccurred in 102 of 1662 patients with COVID-19 admitted in the non-ICU. A continuity correction of 0.001 was utilized in studies with no event. P value for heterogeneity was less than 0.001. |
| Overall (I^2 = 94.08%, p = 0.00)  Study  Mazzaccaro (2020)  Dubois-Silva (2020)  Xu (2020)  Zhang (2020)  Wang (2020)  Logigiani (2020)  Mestre-Gómez (2020)  Middeldorp (2020)  Criel (2020)  Demelo-Rodriguez (2020)  0.01 (0.01, 0.02)  ES (95% CI)  0.06 (0.01, 0.21)  0.00 (0.00, 0.02)  0.01 (0.00, 0.04)  0.42 (0.34, 0.50)  0.00 (0.00, 0.14)  0.01 (0.00, 0.03)  0.00 (0.00, 0.02)  0.02 (0.00, 0.06)  0.04 (0.00, 0.13)  0.12 (0.08, 0.17)  0.01 (0.01, 0.02)  0.06 (0.01, 0.21)  0.00 (0.00, 0.02)  0.01 (0.00, 0.04)  0.42 (0.34, 0.50)  0.00 (0.00, 0.14)  0.01 (0.00, 0.03)  0.00 (0.00, 0.02)  0.02 (0.00, 0.06)  0.04 (0.00, 0.13)  0.12 (0.08, 0.17)      -1.5  -1  -.5  0  .5  1 |
| Figure S9 Forest plot of subgroup analysis of the incidences of VTE in the non-ICU setting based on western or eastern countries. There were 7 studies from western and 3 from eastern countries. VTE events occurred in 103 of 1355 patients from the western coutries and in 68 of 307 patients from the the eastern coutries. P value for heterogeneity between subgroups was 0.716. |
|  |
| 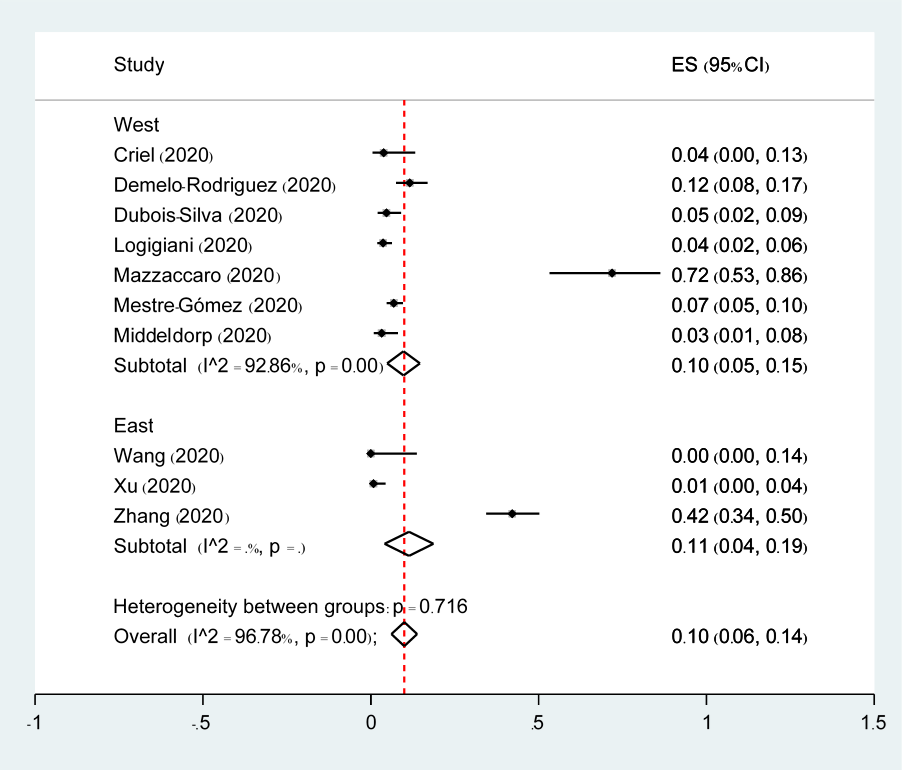 |
| Figure S10 Forest plot of subgroup analysis of the incidences of VTE from clinical studies in the non-ICU setting based on CUS screening. There were 2 studies with no CUS screening and 8 studies with CUS screening. VTE were found in 12 of 327 patients with no CUS screening and in 97 of 680 patients with CUS screening. P value for heterogeneity between subgroups was 0.007. |
| 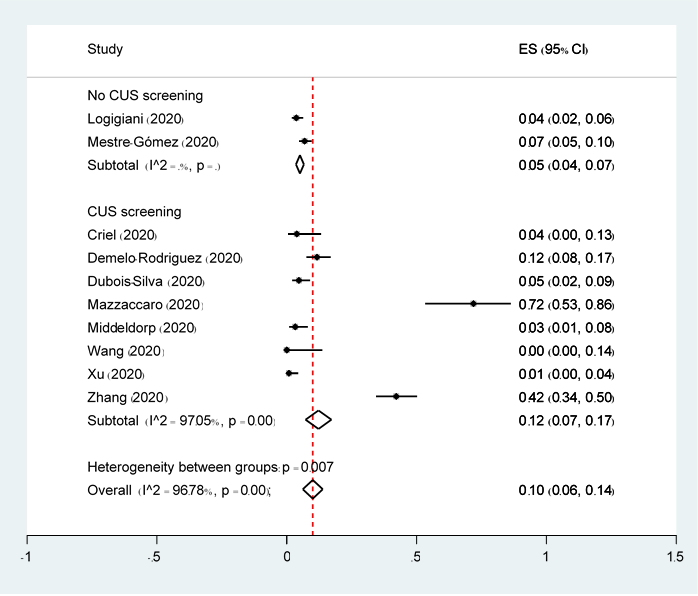 |

| Figure S11 Forest plot of studies showed the pooled incidence of arterial thrombosis from clinical studies in the ICU setting. The analysis included 7 clinical studies. Arterial thrombosis occurred in 30 of 713 patients with COVID-19 admitted in the ICU. P value for heterogeneity was 0.4. |
| --- |
|  |

| Figure S12 Forest plot of studies showed the pooled incidence of arterial thrombosis from clinical studies in the non-ICU setting. The analysis included 2 clinical studies. Arterial thrombosis occurred in 10 of 453 patients with COVID-19 admitted in the non-ICU. |
| --- |
|  |

| Figure S13 Forest plot of the incidences of VTE with death from clinical studies in the ICU setting. The analysis included 6 clinical studies. VTE with death occurred in 48 of 750 patients with COVID-19 admitted in the ICU. P value for heterogeneity was 0.02. |
| --- |
|  |

| Figure S14 Forest plot of studies showed the pooled incidence of VTE from imaging studies. The analysis included 8 imaging studies. VTE was found in 261 of 949 imaging perfomed in patients with COVID-19 requiring hospitalization. P value for heterogeneity was less than 0.001. |
| --- |
|  |

| Figure S15 Forest plot of the incidences of PE from imaging studies focusing on CTPA. The analysis included 6 imaging studies focusing on PE. PE was found in 178 of 712 imaging perfomed in patients with COVID-19 requiring hospitalization. P value for heterogeneity was 0.13. |
| --- |
|  |

| Figure S16 Forest plot of the incidences of DVT from imaging studies focusing on CUS. The analysis included 2 imaging studies focusing on CUS. DVT was found in 16 of 94 imaging perfomed in patients with COVID-19 requiring hospitalization. |
| --- |
|  |

Figure S17 Forest plot of subgroup analysis of the incidences of PE from clinical studies in the ICU setting based on country.


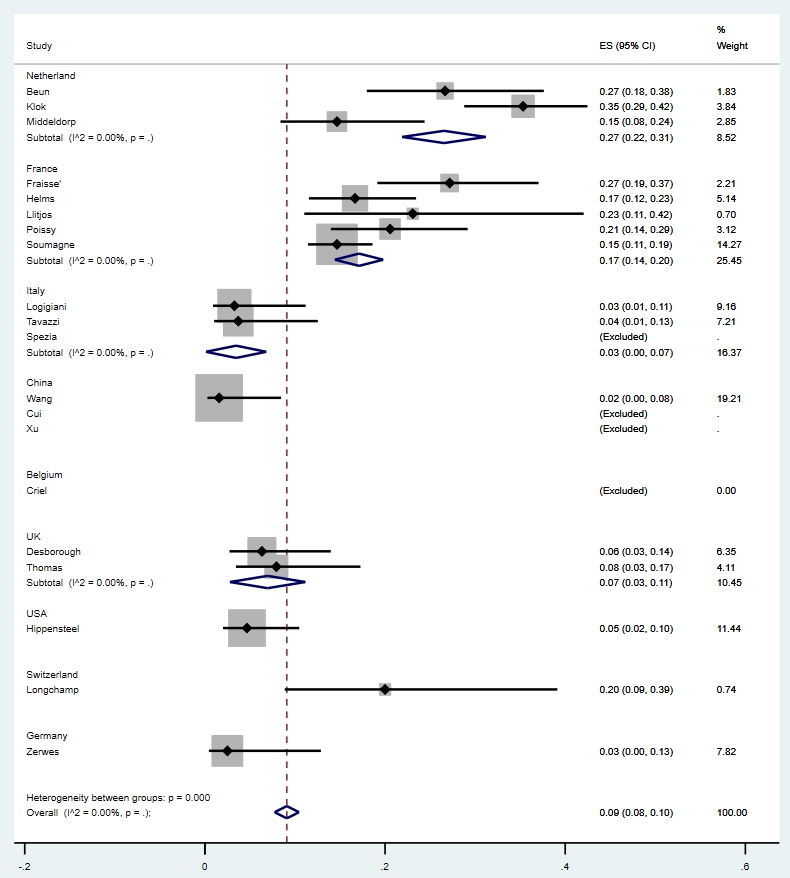

Supplement: Supplementary file 1 — Additional file 1. [file 12959_2020_248_MOESM1_ESM.docx]
